# Minimally Invasive Spinal Surgery

**DOI:** 10.1155/2016/5048659

**Published:** 2016-09-08

**Authors:** Jin-Sung Kim, Roger Härtl, H. Michael Mayer

**Affiliations:** ^1^Spine Center, Department of Neurosurgery, Seoul St. Mary's Hospital, The Catholic University of Korea, Seoul, Republic of Korea; ^2^Weill Cornell Brain and Spine Center, New York, NY, USA; ^3^Paracelsus Medical University, Salzburg, Austria; ^4^Spine Center, Schön Klinik München Harlaching, Munich, Germany

Minimally invasive approaches in spinal surgery are gaining popularity with some evidence in the literature supporting their value.

Significant progress in Minimally Invasive Spinal Surgery (MISS) has been seen in endoscopic spine surgery, minimally invasive fusion, and image-guided spinal surgery. It decreases the incidence of complications and approach-related morbidity and mortality associated with conventional open surgery.

Not only can MISS minimize injury to paraspinal back muscles, connective tissues, and joints but it can also decrease the amount of bleeding, infection, postoperative pain, and hospital stay.

Hence, there has been widespread adoption of MISS techniques, which can be beneficial for various kinds of spinal diseases. In this special issue on Minimally Invasive Spinal Surgery, we have selected high quality papers that highlight several of the benefits of MISS.

With the 7 papers in this issue we cover many of the challenging areas in MISS; three are dealing with image-guided spinal surgery, three are about minimally invasive lumbar interbody fusion including minimally invasive instrumentation for thoracolumbar fractures, one is on a minimally invasive decompressive technique, one is modified technique of laminoplasty, and one is about the novel use of an ultrasonic bone shaver in spine surgery.

H.-S. Kim and D.-H. Heo applied MISS for thoracolumbar fracture associated with Kummell's osteonecrosis and reported advantages of short segment percutaneous pedicle screw fixation with polymethylmethacrylate augmentation.

K.-T. Yeh et al. performed modified expansive open-door laminoplasty (MEOLP) for patients with multilevel cervical spondylotic myelopathy. They concluded that the MEOLP method was found to provide satisfactory medium-term results by preserving muscles and the nuchal ligament attached to the C7 spinous processes, minimizing injury of paraspinal extensor musculature, reducing facet joint violation, and ensuring adequate lamina.

H. M. Mayer and F. Heider also employed minimally invasive Slalom (microsurgical crossover) decompression for multilevel degenerative lumbar stenosis. They reported that minimally invasive decompressive surgery reduces intraoperative blood loss, soft tissue trauma, operative time, infection rates, and hospital stay.

P. Stavrinou et al. retrospectively reviewed 10 patients who underwent transtubular extraforaminal decompression of the L5 nerve root at the lumbosacral junction with navigation. They concluded that navigation is of particular advantage in cases of foraminal stenosis where it allows optimizing the approach angle and amount of bony resection required for successful decompression.

P. Nunley et al. reported neurological complications after lateral transpsoas approach to anterior interbody fusion with a novel flat blade spine-fixed retractor. Using a new retractor system, they observed an 11.1% neurologic complication rate in lateral lumbar interbody fusion procedures. There was resolution of symptoms for most patients by 12-month follow-up, with only 2% of patients with residual symptoms.

T. Kim et al. reported the excellent clinical outcome of intraoperative image-guided navigation. They emphasized that spinal surgery based on O-arm navigation favors it as a good option for spinal instrumentation.

D. B. Hazer et al. reported technical tips of the application of ultrasonic bone shaver in spine surgery experience in 307 patients.

This special issue on Minimally Invasive Spinal Surgery provides valuable information including surgical “tips and pearls” on emerging MIS surgical techniques. Even though all articles were accepted after rigorous blinded reviews by experts in minimally invasive spinal surgical fields, we realize that the level of scientific evidence may be low in some areas. Therefore, we cordially invite the readers to read each article with an open and critical mind.

*Jin-Sung Kim* *Roger Härtl* *H. Michael Mayer*

